# Supplementary material for: Modeling glioblastoma heterogeneity as a dynamic network of cell states
Source: Mol Syst Biol. 2021 Sep 16;17(9):e10105. doi: 10.15252/msb.202010105 (PMC8444284; doi:10.15252/msb.202010105)
Supplement: Supplementary file 6 — Source Data for Figure 5 [file MSB-17-e10105-s004.zip › Figure5A_sourcedata/GSEA_3071/nosigdb_stateIII.GseaPreranked.1622795155562/ALMSTEDT_VGR_DIFF.html]

Details for gene set ALMSTEDT\_VGR\_DIFF[GSEA]

|  || Dataset | state33071 |
| Phenotype | NoPhenotypeAvailable |
| Upregulated in class | na\_neg |
| GeneSet | ALMSTEDT\_VGR\_DIFF |
| Enrichment Score (ES) | -0.39241055 |
| Normalized Enrichment Score (NES) | -2.8169951 |
| Nominal p-value | 0.0 |
| FDR q-value | 0.0 |
| FWER p-Value | 0.0 |
Table: GSEA Results Summary

  

Fig 1: Enrichment plot: ALMSTEDT\_VGR\_DIFF      
 Profile of the Running ES Score & Positions of GeneSet Members on the Rank Ordered List

  

| PROBE | GENE SYMBOL | GENE\_TITLE | RANK IN GENE LIST | RANK METRIC SCORE | RUNNING ES | CORE ENRICHMENT || 1 | HSPB1 |  |  | 4 | 0.650 | 0.0034 | No |
| 2 | RAB13 |  |  | 18 | 0.539 | 0.0036 | No |
| 3 | POLR2L |  |  | 38 | 0.482 | 0.0016 | No |
| 4 | RHOC |  |  | 92 | 0.414 | -0.0101 | No |
| 5 | MYL12B |  |  | 164 | 0.365 | -0.0272 | No |
| 6 | RANBP1 |  |  | 221 | 0.328 | -0.0404 | No |
| 7 | LGALS1 |  |  | 230 | 0.323 | -0.0404 | No |
| 8 | LSM4 |  |  | 268 | 0.303 | -0.0485 | No |
| 9 | CSRP1 |  |  | 345 | 0.279 | -0.0676 | No |
| 10 | HK2 |  |  | 432 | -0.250 | -0.0896 | No |
| 11 | FILIP1L |  |  | 454 | -0.252 | -0.0937 | No |
| 12 | FZD7 |  |  | 457 | -0.252 | -0.0925 | No |
| 13 | PLOD2 |  |  | 521 | -0.255 | -0.1081 | No |
| 14 | LITAF |  |  | 582 | -0.259 | -0.1229 | No |
| 15 | TUBB3 |  |  | 610 | -0.260 | -0.1285 | No |
| 16 | MAGT1 |  |  | 611 | -0.260 | -0.1267 | No |
| 17 | CTSO |  |  | 615 | -0.260 | -0.1258 | No |
| 18 | SIX4 |  |  | 617 | -0.260 | -0.1242 | No |
| 19 | EYA1 |  |  | 620 | -0.261 | -0.1230 | No |
| 20 | OLFML2A |  |  | 635 | -0.261 | -0.1250 | No |
| 21 | TM9SF2 |  |  | 664 | -0.263 | -0.1309 | No |
| 22 | PXDN |  |  | 666 | -0.263 | -0.1294 | No |
| 23 | FLRT2 |  |  | 676 | -0.263 | -0.1301 | No |
| 24 | ELAVL2 |  |  | 695 | -0.264 | -0.1332 | No |
| 25 | KIF1A |  |  | 719 | -0.266 | -0.1377 | No |
| 26 | SHROOM3 |  |  | 720 | -0.266 | -0.1359 | No |
| 27 | CRABP2 |  |  | 739 | -0.266 | -0.1390 | No |
| 28 | PLXDC2 |  |  | 759 | -0.267 | -0.1424 | No |
| 29 | THSD7A |  |  | 767 | -0.268 | -0.1425 | No |
| 30 | GNS |  |  | 769 | -0.268 | -0.1409 | No |
| 31 | DNAJC3 |  |  | 780 | -0.268 | -0.1418 | No |
| 32 | PLAGL1 |  |  | 783 | -0.268 | -0.1405 | No |
| 33 | PLPPR5 |  |  | 824 | -0.271 | -0.1496 | No |
| 34 | MSI2 |  |  | 848 | -0.272 | -0.1541 | No |
| 35 | PKIA |  |  | 854 | -0.272 | -0.1536 | No |
| 36 | HES6 |  |  | 860 | -0.272 | -0.1531 | No |
| 37 | ACTN1 |  |  | 879 | -0.273 | -0.1562 | No |
| 38 | ID3 |  |  | 880 | -0.273 | -0.1543 | No |
| 39 | FUCA2 |  |  | 884 | -0.274 | -0.1532 | No |
| 40 | STEAP1 |  |  | 912 | -0.275 | -0.1588 | No |
| 41 | NBEA |  |  | 913 | -0.275 | -0.1569 | No |
| 42 | ASPH |  |  | 965 | -0.278 | -0.1690 | No |
| 43 | KLF4 |  |  | 973 | -0.278 | -0.1690 | No |
| 44 | SSR1 |  |  | 993 | -0.279 | -0.1723 | No |
| 45 | PRRX1 |  |  | 1032 | -0.281 | -0.1809 | No |
| 46 | BOC |  |  | 1034 | -0.281 | -0.1792 | No |
| 47 | NPTN |  |  | 1054 | -0.282 | -0.1825 | No |
| 48 | SLC38A2 |  |  | 1055 | -0.282 | -0.1806 | No |
| 49 | AUTS2 |  |  | 1056 | -0.282 | -0.1786 | No |
| 50 | NAP1L5 |  |  | 1067 | -0.283 | -0.1794 | No |
| 51 | LAMP1 |  |  | 1072 | -0.283 | -0.1785 | No |
| 52 | WWTR1 |  |  | 1080 | -0.284 | -0.1785 | No |
| 53 | PHYHIPL |  |  | 1087 | -0.284 | -0.1782 | No |
| 54 | FOXO3 |  |  | 1088 | -0.284 | -0.1762 | No |
| 55 | MYEF2 |  |  | 1089 | -0.284 | -0.1742 | No |
| 56 | LPP |  |  | 1097 | -0.284 | -0.1742 | No |
| 57 | EMILIN1 |  |  | 1099 | -0.285 | -0.1725 | No |
| 58 | GPC6 |  |  | 1101 | -0.285 | -0.1708 | No |
| 59 | FOXM1 |  |  | 1102 | -0.285 | -0.1688 | No |
| 60 | ATCAY |  |  | 1109 | -0.285 | -0.1685 | No |
| 61 | DDOST |  |  | 1114 | -0.285 | -0.1676 | No |
| 62 | LAMC1 |  |  | 1120 | -0.286 | -0.1670 | No |
| 63 | TFPI |  |  | 1141 | -0.287 | -0.1706 | No |
| 64 | TMEM108 |  |  | 1200 | -0.290 | -0.1846 | No |
| 65 | SPATA20 |  |  | 1208 | -0.291 | -0.1845 | No |
| 66 | PLSCR4 |  |  | 1223 | -0.291 | -0.1864 | No |
| 67 | EEF1A2 |  |  | 1237 | -0.292 | -0.1879 | No |
| 68 | FSTL1 |  |  | 1254 | -0.293 | -0.1903 | No |
| 69 | RUFY3 |  |  | 1257 | -0.293 | -0.1888 | No |
| 70 | NUSAP1 |  |  | 1262 | -0.293 | -0.1879 | No |
| 71 | TIAM1 |  |  | 1276 | -0.294 | -0.1895 | No |
| 72 | PLEKHH2 |  |  | 1282 | -0.294 | -0.1888 | No |
| 73 | GABRB3 |  |  | 1291 | -0.295 | -0.1890 | No |
| 74 | RNF144A |  |  | 1311 | -0.295 | -0.1922 | No |
| 75 | ATP2B4 |  |  | 1347 | -0.297 | -0.1998 | No |
| 76 | GDPD1 |  |  | 1375 | -0.299 | -0.2052 | No |
| 77 | LAPTM4A |  |  | 1391 | -0.299 | -0.2072 | No |
| 78 | IGF2R |  |  | 1432 | -0.302 | -0.2162 | No |
| 79 | GGH |  |  | 1439 | -0.302 | -0.2157 | No |
| 80 | FIBIN |  |  | 1458 | -0.304 | -0.2186 | No |
| 81 | PPP1R9A |  |  | 1472 | -0.304 | -0.2201 | No |
| 82 | CTSC |  |  | 1511 | -0.306 | -0.2285 | No |
| 83 | WLS |  |  | 1517 | -0.307 | -0.2277 | No |
| 84 | WNT5A |  |  | 1531 | -0.308 | -0.2292 | No |
| 85 | SLC10A4 |  |  | 1561 | -0.310 | -0.2350 | No |
| 86 | ITPRIPL2 |  |  | 1563 | -0.310 | -0.2332 | No |
| 87 | KLHL13 |  |  | 1566 | -0.310 | -0.2316 | No |
| 88 | FAM102B |  |  | 1567 | -0.310 | -0.2294 | No |
| 89 | ANTXR1 |  |  | 1578 | -0.311 | -0.2300 | No |
| 90 | LRRTM2 |  |  | 1585 | -0.311 | -0.2295 | No |
| 91 | MYBL2 |  |  | 1587 | -0.311 | -0.2276 | No |
| 92 | PLOD3 |  |  | 1588 | -0.311 | -0.2255 | No |
| 93 | KIDINS220 |  |  | 1589 | -0.311 | -0.2233 | No |
| 94 | KLF6 |  |  | 1596 | -0.312 | -0.2228 | No |
| 95 | MAML2 |  |  | 1603 | -0.312 | -0.2223 | No |
| 96 | BEX2 |  |  | 1617 | -0.313 | -0.2237 | No |
| 97 | PTGER4 |  |  | 1628 | -0.314 | -0.2243 | No |
| 98 | RTN2 |  |  | 1675 | -0.317 | -0.2348 | No |
| 99 | FSD1 |  |  | 1677 | -0.317 | -0.2329 | No |
| 100 | GAL |  |  | 1679 | -0.317 | -0.2310 | No |
| 101 | DUSP4 |  |  | 1703 | -0.319 | -0.2351 | No |
| 102 | GNG4 |  |  | 1706 | -0.319 | -0.2335 | No |
| 103 | PDK1 |  |  | 1723 | -0.320 | -0.2356 | No |
| 104 | SRPX |  |  | 1747 | -0.322 | -0.2398 | No |
| 105 | PEG3 |  |  | 1754 | -0.322 | -0.2392 | No |
| 106 | NUF2 |  |  | 1755 | -0.322 | -0.2370 | No |
| 107 | AEBP1 |  |  | 1763 | -0.322 | -0.2367 | No |
| 108 | COL12A1 |  |  | 1764 | -0.322 | -0.2344 | No |
| 109 | FGFR1 |  |  | 1770 | -0.323 | -0.2336 | No |
| 110 | ACVR1B |  |  | 1800 | -0.324 | -0.2393 | No |
| 111 | ZNF91 |  |  | 1803 | -0.325 | -0.2376 | No |
| 112 | TTC8 |  |  | 1815 | -0.325 | -0.2384 | No |
| 113 | FKBP14 |  |  | 1860 | -0.327 | -0.2483 | No |
| 114 | ADAM9 |  |  | 1870 | -0.328 | -0.2485 | No |
| 115 | ZNF711 |  |  | 1936 | -0.331 | -0.2642 | No |
| 116 | ABLIM1 |  |  | 1940 | -0.332 | -0.2627 | No |
| 117 | UBE2C |  |  | 1942 | -0.332 | -0.2607 | No |
| 118 | SPRY4 |  |  | 1970 | -0.334 | -0.2658 | No |
| 119 | ITGA4 |  |  | 1986 | -0.334 | -0.2676 | No |
| 120 | RNFT2 |  |  | 1990 | -0.335 | -0.2661 | No |
| 121 | KIAA1211 |  |  | 2008 | -0.335 | -0.2685 | No |
| 122 | ANKRD46 |  |  | 2028 | -0.337 | -0.2714 | No |
| 123 | TIMP1 |  |  | 2038 | -0.337 | -0.2716 | No |
| 124 | FKBP1B |  |  | 2065 | -0.339 | -0.2764 | No |
| 125 | PPIB |  |  | 2111 | -0.342 | -0.2865 | No |
| 126 | RAB6B |  |  | 2129 | -0.344 | -0.2888 | No |
| 127 | RRM2 |  |  | 2147 | -0.345 | -0.2911 | No |
| 128 | MXI1 |  |  | 2182 | -0.347 | -0.2981 | No |
| 129 | TM4SF1 |  |  | 2186 | -0.348 | -0.2965 | No |
| 130 | ZNF704 |  |  | 2210 | -0.350 | -0.3004 | No |
| 131 | SASH1 |  |  | 2231 | -0.351 | -0.3035 | No |
| 132 | RNF165 |  |  | 2247 | -0.352 | -0.3052 | No |
| 133 | INSIG1 |  |  | 2266 | -0.354 | -0.3077 | No |
| 134 | MCM2 |  |  | 2303 | -0.357 | -0.3152 | No |
| 135 | GDF15 |  |  | 2323 | -0.359 | -0.3179 | No |
| 136 | SLC38A6 |  |  | 2344 | -0.361 | -0.3210 | No |
| 137 | GJA1 |  |  | 2362 | -0.362 | -0.3231 | No |
| 138 | ENO2 |  |  | 2366 | -0.363 | -0.3215 | No |
| 139 | FAM129A |  |  | 2371 | -0.363 | -0.3200 | No |
| 140 | KDM5B |  |  | 2402 | -0.365 | -0.3258 | No |
| 141 | EDEM1 |  |  | 2411 | -0.366 | -0.3255 | No |
| 142 | GRN |  |  | 2426 | -0.368 | -0.3268 | No |
| 143 | CD44 |  |  | 2471 | -0.371 | -0.3364 | No |
| 144 | PRCP |  |  | 2489 | -0.372 | -0.3385 | No |
| 145 | PDIA6 |  |  | 2505 | -0.373 | -0.3400 | No |
| 146 | CHGB |  |  | 2506 | -0.374 | -0.3374 | No |
| 147 | MMP2 |  |  | 2527 | -0.375 | -0.3404 | No |
| 148 | FBN2 |  |  | 2528 | -0.375 | -0.3378 | No |
| 149 | SDC2 |  |  | 2537 | -0.376 | -0.3374 | No |
| 150 | IQGAP2 |  |  | 2538 | -0.376 | -0.3347 | No |
| 151 | ROBO1 |  |  | 2546 | -0.377 | -0.3341 | No |
| 152 | LAMB1 |  |  | 2556 | -0.378 | -0.3339 | No |
| 153 | FLNA |  |  | 2559 | -0.378 | -0.3319 | No |
| 154 | QSOX1 |  |  | 2574 | -0.380 | -0.3331 | No |
| 155 | TMTC4 |  |  | 2583 | -0.380 | -0.3327 | No |
| 156 | HEY1 |  |  | 2593 | -0.381 | -0.3325 | No |
| 157 | TACC2 |  |  | 2615 | -0.383 | -0.3357 | No |
| 158 | CKAP4 |  |  | 2664 | -0.387 | -0.3462 | No |
| 159 | RTN1 |  |  | 2674 | -0.388 | -0.3460 | No |
| 160 | ID1 |  |  | 2685 | -0.389 | -0.3461 | No |
| 161 | ST3GAL6 |  |  | 2709 | -0.391 | -0.3497 | No |
| 162 | ACTA2 |  |  | 2721 | -0.393 | -0.3500 | No |
| 163 | C11orf95 |  |  | 2741 | -0.394 | -0.3526 | No |
| 164 | LMAN1 |  |  | 2743 | -0.394 | -0.3501 | No |
| 165 | CBLB |  |  | 2745 | -0.394 | -0.3476 | No |
| 166 | KIF21A |  |  | 2786 | -0.398 | -0.3559 | No |
| 167 | ATP1B1 |  |  | 2865 | -0.405 | -0.3747 | No |
| 168 | DDR2 |  |  | 2874 | -0.406 | -0.3741 | No |
| 169 | ELAVL3 |  |  | 2898 | -0.408 | -0.3776 | No |
| 170 | FAM171B |  |  | 2900 | -0.408 | -0.3750 | No |
| 171 | SVIL |  |  | 2912 | -0.409 | -0.3752 | No |
| 172 | CTSB |  |  | 2916 | -0.410 | -0.3732 | No |
| 173 | SERPINH1 |  |  | 2921 | -0.410 | -0.3715 | No |
| 174 | ITGAV |  |  | 2942 | -0.414 | -0.3741 | No |
| 175 | PRSS12 |  |  | 2960 | -0.415 | -0.3760 | No |
| 176 | EGR1 |  |  | 2961 | -0.415 | -0.3731 | No |
| 177 | ASRGL1 |  |  | 2986 | -0.418 | -0.3768 | No |
| 178 | EMP1 |  |  | 2998 | -0.420 | -0.3769 | No |
| 179 | MRC2 |  |  | 3032 | -0.424 | -0.3831 | No |
| 180 | PPIC |  |  | 3045 | -0.425 | -0.3835 | No |
| 181 | OLFML2B |  |  | 3050 | -0.425 | -0.3816 | No |
| 182 | NMNAT2 |  |  | 3084 | -0.429 | -0.3878 | No |
| 183 | SMAD3 |  |  | 3091 | -0.430 | -0.3865 | No |
| 184 | JAM3 |  |  | 3096 | -0.430 | -0.3846 | No |
| 185 | TMOD2 |  |  | 3114 | -0.433 | -0.3863 | No |
| 186 | PDIA4 |  |  | 3130 | -0.435 | -0.3874 | No |
| 187 | ADAM19 |  |  | 3132 | -0.435 | -0.3846 | No |
| 188 | SGK1 |  |  | 3150 | -0.437 | -0.3863 | No |
| 189 | TSC22D3 |  |  | 3157 | -0.439 | -0.3849 | No |
| 190 | RALGDS |  |  | 3164 | -0.440 | -0.3835 | No |
| 191 | PTGFRN |  |  | 3166 | -0.440 | -0.3808 | No |
| 192 | ADAMTS5 |  |  | 3188 | -0.441 | -0.3835 | No |
| 193 | HSPA5 |  |  | 3193 | -0.442 | -0.3815 | No |
| 194 | SYT1 |  |  | 3200 | -0.442 | -0.3801 | No |
| 195 | SOX11 |  |  | 3205 | -0.443 | -0.3782 | No |
| 196 | HTRA1 |  |  | 3242 | -0.450 | -0.3850 | No |
| 197 | PTPRG |  |  | 3246 | -0.451 | -0.3827 | No |
| 198 | TRAM2 |  |  | 3247 | -0.451 | -0.3796 | No |
| 199 | FZD1 |  |  | 3256 | -0.452 | -0.3786 | No |
| 200 | CD63 |  |  | 3258 | -0.452 | -0.3758 | No |
| 201 | GATA3 |  |  | 3260 | -0.453 | -0.3729 | No |
| 202 | SLC35G2 |  |  | 3287 | -0.457 | -0.3769 | No |
| 203 | GAP43 |  |  | 3308 | -0.462 | -0.3792 | No |
| 204 | ITM2B |  |  | 3314 | -0.463 | -0.3774 | No |
| 205 | SBK1 |  |  | 3334 | -0.468 | -0.3794 | No |
| 206 | EDNRA |  |  | 3351 | -0.471 | -0.3806 | No |
| 207 | MEST |  |  | 3363 | -0.473 | -0.3803 | No |
| 208 | NAPB |  |  | 3389 | -0.479 | -0.3839 | No |
| 209 | OLFM1 |  |  | 3398 | -0.480 | -0.3828 | No |
| 210 | GDAP1 |  |  | 3420 | -0.484 | -0.3852 | No |
| 211 | EGFR |  |  | 3433 | -0.488 | -0.3852 | No |
| 212 | ITGA10 |  |  | 3442 | -0.491 | -0.3840 | No |
| 213 | SLC30A1 |  |  | 3458 | -0.495 | -0.3847 | No |
| 214 | ZFP36L1 |  |  | 3487 | -0.503 | -0.3889 | Yes |
| 215 | CYFIP2 |  |  | 3493 | -0.505 | -0.3868 | Yes |
| 216 | GAS1 |  |  | 3494 | -0.505 | -0.3833 | Yes |
| 217 | DPYSL3 |  |  | 3498 | -0.507 | -0.3806 | Yes |
| 218 | LRRC8C |  |  | 3500 | -0.507 | -0.3774 | Yes |
| 219 | OLFML3 |  |  | 3503 | -0.509 | -0.3744 | Yes |
| 220 | TRIL |  |  | 3505 | -0.510 | -0.3711 | Yes |
| 221 | TSPAN7 |  |  | 3528 | -0.520 | -0.3736 | Yes |
| 222 | F2R |  |  | 3538 | -0.523 | -0.3724 | Yes |
| 223 | LMO3 |  |  | 3547 | -0.526 | -0.3710 | Yes |
| 224 | DKK1 |  |  | 3556 | -0.530 | -0.3695 | Yes |
| 225 | SOX9 |  |  | 3560 | -0.531 | -0.3667 | Yes |
| 226 | NRP1 |  |  | 3562 | -0.532 | -0.3633 | Yes |
| 227 | CALU |  |  | 3566 | -0.533 | -0.3604 | Yes |
| 228 | PARP6 |  |  | 3568 | -0.534 | -0.3570 | Yes |
| 229 | CKB |  |  | 3572 | -0.536 | -0.3541 | Yes |
| 230 | SERPINE2 |  |  | 3573 | -0.537 | -0.3504 | Yes |
| 231 | ITGB1 |  |  | 3574 | -0.537 | -0.3466 | Yes |
| 232 | ATP2B1 |  |  | 3580 | -0.539 | -0.3443 | Yes |
| 233 | ALK |  |  | 3585 | -0.540 | -0.3416 | Yes |
| 234 | LRRC17 |  |  | 3590 | -0.543 | -0.3390 | Yes |
| 235 | TUBB2B |  |  | 3594 | -0.544 | -0.3360 | Yes |
| 236 | RRBP1 |  |  | 3595 | -0.545 | -0.3322 | Yes |
| 237 | NES |  |  | 3596 | -0.545 | -0.3285 | Yes |
| 238 | THBS1 |  |  | 3599 | -0.547 | -0.3252 | Yes |
| 239 | VCL |  |  | 3603 | -0.551 | -0.3222 | Yes |
| 240 | SPRY1 |  |  | 3605 | -0.552 | -0.3187 | Yes |
| 241 | DACH1 |  |  | 3607 | -0.553 | -0.3151 | Yes |
| 242 | TPBG |  |  | 3638 | -0.570 | -0.3194 | Yes |
| 243 | MAP2 |  |  | 3640 | -0.572 | -0.3158 | Yes |
| 244 | SHC3 |  |  | 3642 | -0.573 | -0.3121 | Yes |
| 245 | F2RL2 |  |  | 3645 | -0.574 | -0.3086 | Yes |
| 246 | KCTD12 |  |  | 3646 | -0.574 | -0.3046 | Yes |
| 247 | DUSP6 |  |  | 3648 | -0.577 | -0.3009 | Yes |
| 248 | DPYSL5 |  |  | 3651 | -0.580 | -0.2974 | Yes |
| 249 | CFH |  |  | 3653 | -0.582 | -0.2937 | Yes |
| 250 | COL4A2 |  |  | 3664 | -0.586 | -0.2924 | Yes |
| 251 | DLX1 |  |  | 3665 | -0.589 | -0.2883 | Yes |
| 252 | CXADR |  |  | 3670 | -0.591 | -0.2853 | Yes |
| 253 | COL6A2 |  |  | 3674 | -0.595 | -0.2820 | Yes |
| 254 | FAT1 |  |  | 3685 | -0.604 | -0.2806 | Yes |
| 255 | SEL1L3 |  |  | 3686 | -0.604 | -0.2764 | Yes |
| 256 | BMP7 |  |  | 3698 | -0.613 | -0.2751 | Yes |
| 257 | ITPR1 |  |  | 3706 | -0.623 | -0.2728 | Yes |
| 258 | PNMA2 |  |  | 3720 | -0.633 | -0.2720 | Yes |
| 259 | GRIA2 |  |  | 3721 | -0.633 | -0.2676 | Yes |
| 260 | COL5A2 |  |  | 3726 | -0.636 | -0.2643 | Yes |
| 261 | SATB1 |  |  | 3732 | -0.644 | -0.2612 | Yes |
| 262 | AKAP12 |  |  | 3740 | -0.660 | -0.2585 | Yes |
| 263 | DLX2 |  |  | 3742 | -0.662 | -0.2542 | Yes |
| 264 | FHOD3 |  |  | 3747 | -0.668 | -0.2507 | Yes |
| 265 | HSP90B1 |  |  | 3757 | -0.681 | -0.2484 | Yes |
| 266 | ARL6IP1 |  |  | 3759 | -0.685 | -0.2440 | Yes |
| 267 | FZD3 |  |  | 3765 | -0.695 | -0.2405 | Yes |
| 268 | TMEFF2 |  |  | 3767 | -0.697 | -0.2360 | Yes |
| 269 | INSM1 |  |  | 3772 | -0.704 | -0.2322 | Yes |
| 270 | SOSTDC1 |  |  | 3773 | -0.705 | -0.2273 | Yes |
| 271 | APP |  |  | 3779 | -0.710 | -0.2237 | Yes |
| 272 | SCG2 |  |  | 3786 | -0.718 | -0.2204 | Yes |
| 273 | CDH11 |  |  | 3789 | -0.721 | -0.2160 | Yes |
| 274 | FBN1 |  |  | 3790 | -0.723 | -0.2110 | Yes |
| 275 | TFAP2B |  |  | 3792 | -0.729 | -0.2062 | Yes |
| 276 | TAGLN3 |  |  | 3793 | -0.729 | -0.2011 | Yes |
| 277 | KIF5C |  |  | 3798 | -0.739 | -0.1971 | Yes |
| 278 | CXCR4 |  |  | 3802 | -0.750 | -0.1927 | Yes |
| 279 | EML6 |  |  | 3806 | -0.755 | -0.1883 | Yes |
| 280 | SPARC |  |  | 3811 | -0.759 | -0.1841 | Yes |
| 281 | BGN |  |  | 3812 | -0.761 | -0.1789 | Yes |
| 282 | NCAN |  |  | 3813 | -0.761 | -0.1736 | Yes |
| 283 | COL5A1 |  |  | 3815 | -0.764 | -0.1686 | Yes |
| 284 | NCAM1 |  |  | 3821 | -0.773 | -0.1646 | Yes |
| 285 | CELF2 |  |  | 3822 | -0.774 | -0.1592 | Yes |
| 286 | IGSF3 |  |  | 3824 | -0.777 | -0.1541 | Yes |
| 287 | BEX1 |  |  | 3825 | -0.779 | -0.1487 | Yes |
| 288 | COL11A1 |  |  | 3828 | -0.788 | -0.1438 | Yes |
| 289 | COL4A1 |  |  | 3829 | -0.791 | -0.1383 | Yes |
| 290 | MIAT |  |  | 3833 | -0.794 | -0.1336 | Yes |
| 291 | TCEAL7 |  |  | 3834 | -0.798 | -0.1281 | Yes |
| 292 | FN1 |  |  | 3850 | -0.842 | -0.1264 | Yes |
| 293 | ASCL1 |  |  | 3852 | -0.843 | -0.1208 | Yes |
| 294 | PROM1 |  |  | 3854 | -0.846 | -0.1152 | Yes |
| 295 | CADM1 |  |  | 3856 | -0.851 | -0.1096 | Yes |
| 296 | COL6A1 |  |  | 3863 | -0.872 | -0.1052 | Yes |
| 297 | SCRG1 |  |  | 3869 | -0.904 | -0.1003 | Yes |
| 298 | TNC |  |  | 3874 | -0.951 | -0.0948 | Yes |
| 299 | NRCAM |  |  | 3881 | -1.009 | -0.0894 | Yes |
| 300 | PTN |  |  | 3884 | -1.022 | -0.0829 | Yes |
| 301 | POSTN |  |  | 3885 | -1.025 | -0.0758 | Yes |
| 302 | CYR61 |  |  | 3887 | -1.037 | -0.0689 | Yes |
| 303 | A2M |  |  | 3890 | -1.053 | -0.0621 | Yes |
| 304 | APOE |  |  | 3891 | -1.056 | -0.0548 | Yes |
| 305 | IGFBP5 |  |  | 3895 | -1.082 | -0.0481 | Yes |
| 306 | COL6A3 |  |  | 3900 | -1.171 | -0.0411 | Yes |
| 307 | COL1A1 |  |  | 3902 | -1.191 | -0.0331 | Yes |
| 308 | NFIA |  |  | 3910 | -1.317 | -0.0259 | Yes |
| 309 | DCX |  |  | 3911 | -1.320 | -0.0168 | Yes |
| 310 | COL3A1 |  |  | 3918 | -1.418 | -0.0086 | Yes |
| 311 | MGP |  |  | 3927 | -1.633 | 0.0006 | Yes |
Table: GSEA details [plain text format]

  

Fig 2: ALMSTEDT\_VGR\_DIFF: Random ES distribution      
 Gene set null distribution of ES for **ALMSTEDT\_VGR\_DIFF**
